# Hand hygiene compliance from an occupational health perspective - the importance of choosing the right handrub to prevent contact dermatitis

**DOI:** 10.1017/ash.2025.156

**Published:** 2025-09-03

**Authors:** Sylvia Teo, Thoon Koh Cheng

**Affiliations:** a Family Medicine ServiceKK Women’s and Children’s Hospital, Singapore

## Abstract

**Objectives:** Hand hygiene is the cornerstone of infection prevention and control. Factors associated with hand hygiene compliance continues to be a key focus of research today. Choosing the ‘right’ alcohol-based hand rub (ABHR) is pivotal in mitigating ICD (irritant contact dermatitis) caused or aggravated by repetitive use of ABHRs. 31% of our healthcare workers (HCW)s reported worsening of their underlying eczema from repetitive use of the incumbent ABHR, whilst 74% experienced either dryness or ICD. In this study, we aim to evaluate two ethanol-based hand rubs against the incumbent hand rub (ethanol and n-propanol mix) to determine if skin tolerability and incidence of ICD can be improved. **Methods:** 500 HCWs from three departments were invited to participate in a survey to evaluate skin tolerability and user’s acceptability of the 1) incumbent ABHR (i-ABHR) (176 responded) and (2) New ABHR 1 (n-ABHR-1) (147 responded), whilst 190 HCWs from one department were invited to trial 3) New ABHR 2 (n-ABHR-2) (87 responded) using the WHO protocol 1 for handrub evaluation over the course of two weeks for each product respectively. **Results:** For skin tolerability assessment, only n-ABHR-2 achieved the product acceptability criteria of ≥75% of the participants choosing scale 5 or higher, whereas the other two ABHRs did not. Only 47.7% felt that i-ABHR provided acceptable moisture content. Product acceptability wise, only n-ABHR-2 achieved the acceptable criteria for all measurements. For n-ABHR-1, 45% experienced ICD or dryness, whilst 9% reported worsening of underlying eczema. For n-ABHR 2, 23% reported ICD whilst 1% experienced worsening of underlying eczema. **Conclusion:** In conclusion, n-ABHR-2 was a more suitable alternative compared to i-ABHR and n-ABHR-1 in terms of acceptability and skin tolerability, particularly for those with underlying eczema. Further investigation is warranted to determine if the low ICD rate could be maintained longer-term to ensure hand hygiene compliance.

